# Bacteriostatic Activity of LLDPE Nanocomposite Embedded with Sol–Gel Synthesized TiO_2_/ZnO Coupled Oxides at Various Ratios

**DOI:** 10.3390/polym10080878

**Published:** 2018-08-06

**Authors:** Khairul Arifah Saharudin, Srimala Sreekantan, Norfatehah Basiron, Yong Ling Khor, Nor Hazliana Harun, Rabiatul Basria S. M. N. Mydin, Hazizan Md Akil, Azman Seeni, Kumaravel Vignesh

**Affiliations:** 1 School of Materials & Mineral Resources Engineering, Engineering Campus, Universiti Sains Malaysia, Nibong Tebal, Pulau Pinang 14300, Malaysia; khairularifah@gmail.com (K.A.S.); misz_teha@yahoo.com (N.B.); kyling9342@gmail.com (Y.L.K.); hazizan@usm.my (H.M.A.); 2 Advanced Medical and Dental Institute, Universiti Sains Malaysia, Bertam, Pulau Pinang 13200, Malaysia; hazlianarun@gmail.com (N.H.H.); rabiatulbasria@usm.my (R.B.S.M.N.M.); 3 Malaysian Institute of Pharmaceuticals and Nutraceuticals (IPHARM), National Institute of Biotechnology Malaysia, Ministry of Science, Technology and Innovation, Bukit Gambir, Gelugor, Pulau Pinang 11700, Malaysia; azmanseeni@usm.my; 4 Department of Environmental Science, School of Science, Institute of Technology Sligo, Ash Lane, F91 YW50 Sligo, Ireland; vignesh134@gmail.com

**Keywords:** LLDPE nanocomposites, TiO_2_/ZnO nanoparticles, bacteriostatic activity, *Staphylococcus aureus*, *Escherichia coli*

## Abstract

Metal oxide-polymer nanocomposite has been proven to have selective bactericidal effects against the main and common pathogens (Gram-positive *Staphylococcus aureus* (*S. aureus*) and Gram-negative *Escherichia coli* (*E. coli*)) that can cause harmful infectious diseases. As such, this study looked into the prospect of using TiO_2_/ZnO with linear low-density polyethylene (LLDPE) to inactivate *S. aureus* and *E. coli*. The physical, structural, chemical, mechanical, and antibacterial properties of the nanocomposite were investigated in detail in this paper. The production of reactive species, such as hydroxyl radicals (^•^OH), holes (h^+^), superoxide anion radicals (O_2_^•^¯), and zinc ion (Zn^2+^), released from the nanocomposite were quantified to elucidate the underlying antibacterial mechanisms. LLDPE/25T75Z with TiO_2_/ZnO (1:3) nanocomposite displayed the best performance that inactivated *S. aureus* and *E. coli* by 95% and 100%, respectively. The dominant reactive active species and the zinc ion release toward the superior antibacterial effect of nanocomposite are discussed. This work does not only offer depiction of the effective element required for antimicrobial biomedical appliances, but also the essential structural characteristics to enhance water uptake to expedite photocatalytic activity of LLDPE/metal oxide nanocomposite for long term application.

## 1. Introduction

Microbial infection on polymer-based biomedical appliance appears to be a major threat within the healthcare industry, as it can cause infectious outbreaks and economic losses. Gram-positive *Staphylococcus aureus* (*S. aureus*) and Gram-negative *Escherichia coli* (*E. coli*) are the two major nosocomial pathogens that can lead to a wide spectrum of infections, including skin and soft tissue infections, surgical site infections (SSI), catheter-related infections, septic shock, pneumonia, endocarditis, bacteremia, and cellulitis [1,2]. Nevertheless, bacterial resistance towards antibiotics and its dissemination have resulted in major health problems, which has led to treatment drawbacks for a substantial number of drugs [3]. With that said, increasing interest has been noted regarding the use of nanostructured materials, such as metal- or metal oxide-embedded polymers, as the antimicrobial substance against pathogens. In fact, numerous techniques have been proposed to integrate antimicrobial properties in a polymer matrix. For instance, incorporation of antimicrobial agents directly into polymers [4] can be performed by coating or adsorbing antimicrobials onto polymer surfaces [5,6], immobilization of antimicrobials to polymers via ion or covalent linkages [7], and the use of polymers that are inherently antimicrobial [8]. Amongst these methods, incorporation of inorganic metal/metal oxide in a polymer has gained priority in a number of studies due to the unique antibacterial activity of metal/metal oxide at low concentration [7], stability in extreme conditions [5,6], and non-toxicity [8,9,10]. As such, varied types of metals (e.g., copper (Cu) [11,12], silver (Ag) [13,14]), and metal oxide nanoparticles (zinc oxide (ZnO) [15,16], titanium dioxide (TiO_2_) [17,18], and copper oxide (CuO) [5,6]) have been amalgamated with polymers to promote antimicrobial activity. Some of the advantages of using metal oxide nanoparticles are their effectiveness against a wide range of pathogens [19], non-toxic, heat resistance [20], high photocatalytic activity [18], and exceptional biocompatibility [21]. Among the numerous types of metal oxides available, TiO_2_ appears to be one of the most versatile antimicrobial agents due to its excellent photo-induced antimicrobial activity over a broad spectrum of microorganisms. The antimicrobial properties of TiO_2_ can be attributed to the production of reactive oxygen species (ROS) (e.g., ^•^OH, O_2_^•^¯, and H_2_O_2_ [22]). These ROS attack toxic microorganisms by adhering to the surface of TiO_2_ and destroying them via oxidative mechanism. Nguyen et al. [23] reported that PE/TiO_2_ nanocomposites films exhibited biocidal properties with an inhibition ratio of 50.4% for *E. coli* and 58.0% for *S. aureus*. Yanez et al. [18] discovered that TiO_2_ nanotubes incorporated with linear low-density polyethylene (LLDPE) matrix displayed biocidal activity approximately 63.5% against *E. coli*. ZnO, which is an alternative to TiO_2_ for biocidal applications [24], has been listed as one of the five zinc compounds generally recognized as safe (GRAS) by the U.S. Food and Drug Administration (FDA) [25]. Teli and Kale described that PET composite fibers with 1% ZnO showed 80% and 75% of antibacterial activities against *S. aureus* and *E. coli* respectively, mainly due to the production of ROS and the accumulation of nanoparticles in the cell membranes [17]. Some reports suggest that the biocidal effect of ZnO is mainly caused by Zn^2+^ ion release, which causes cell death via disruption of metabolic pathways and protein synthesis [26]. Nonetheless, complete 100% inhibition was not achieved using single-phase metal oxides due to several limitations, such as poor light harvesting efficiency, poor photoresponse, inefficient charge transport, and separation [27]. According to Cai et al., [28] the use of coupled metal oxide nanoparticles emerged as the best choice to address these limitations. The coupled TiO_2_/ZnO nanoparticles can increase quantum efficiency, primarily because coupled semiconductors have the ability to enhance the rate of separation for photo-induced charge carriers in photocatalyst [28]. Further increment of light harvesting efficiency and antimicrobial efficacy could be attained through the formation of heterojunction between TiO_2_ and ZnO. This improves the charge transport and enhances the charge carrier separation, in comparison to bare metal oxide [29,30,31]. Nevertheless, most studies are concerned only with nanoparticles [26,32,33,34], while studies related to antimicrobial efficacy of nanoparticle-embedded polymer matrix are scarce. To the best of the authors’ knowledge, no comprehensive report is available pertaining to the structural aspects and the antimicrobial efficacy of TiO_2_/ZnO-embedded LLDPE polymer matrix against *S. aureus* and *E. coli*. Furthermore, the release of reactive species and their antimicrobial mechanism have yet to be explored in-depth. Hence, this present work presents the synthesis of LLDPE embedded with TiO_2_/ZnO nanocomposite via wet casting method, wherein mechanical property, thermal analysis, scavenger test, ion release, and antimicrobial activity are investigated in detail. LLDPE polymer has been vastly used due to its excellent flexibility, mechanical strength, and durability [35]. This study proposes the antimicrobial mechanisms of the nanocomposite against *S. aureus* and *E. coli*.

## 2. Materials and Methods 

### 2.1. Materials

All chemicals used in this study were of analytical grade and purchased from Sigma Aldrich (Darmstadt, Germany). LLDPE pellets were obtained from Lotte Chemical Titan (M) Sdn. Bhd. (Kuala Lumpur, Malaysia). The chemicals were used as received without further purification.

### 2.2. Synthesis of TiO_2_/ZnO Nanocomposite

TiO_2_/ZnO nanocomposite prepared in different ratios (1:1, 1:3, 3:1) had been synthesized via sol–gel method. ZnO sol was prepared by adding 0.114 mole of zinc acetate dehydrate (ZAD) into ethanol and stirred at 70 °C for 5 min. Then, the mixture was stirred at room temperature for another 30 min. Subsequently, 2 mL of deionized water was added dropwise, and the stirring was continued for another 5 h. The suspension was centrifuged at 3000 rpm for 15 min. The precipitate was washed with deionized water for several times, and then dried in an oven overnight at 80 °C. The dried sample was pulverized with pestle and mortar and then calcined in air at 500 °C for 2 h. The sample was designated as 100Z.

As for TiO_2_ sol, titanium isopropoxide (TTIP) was mixed in ethanol with a volume ratio of 1:4 and stirred for 30 min. Next, deionized water with a volume ratio of H_2_O:precursor solution = 1:100 was added dropwise into the TTIP mixture and stirred for 3 h. The suspension was centrifuged, washed, dried, and calcined. This sample was designated as 100T. In preparing TiO_2_/ZnO nanocomposite, ZnO and TiO_2_ sols were synthesized separately by adhering to the aforementioned procedure, and then mixed according to their molar ratios (TiO_2_/ZnO = 1:3, 1:1, 3:1). The samples were designated as 25T75Z, 50T50Z, and 75T25Z for TiO_2_/ZnO ratios of 1:3, 1:1, and 3:1, respectively.

### 2.3. Fabrication of LLDPE/Metal Oxide Nanocomposite

The nanocomposites were prepared using the wet casting method. LLDPE pellets (1 g) were dissolved in 15 ml of 1,2-dichlorobenzene at 70 °C under continuous stirring for 10 min (solution A). After that, 5 wt % of metal oxide nanoparticles were added into 10 mL of 1,2-dichlorobenzene and sonicated for 3 min (solution B). Next, solution B was added dropwise into solution A under continuous stirring for 10 min. The mixture was poured into 90 mm diameter petri dish and oven dried at 80 °C for 18 h to generate LLDPE/metal oxide polymer film. Pure LLDPE polymer films were synthesized by adhering to the same procedure without including metal oxide. The samples were designated as LLDPE, LLDPE/100T, LLDPE/100Z, LLDPE/25T75Z, LLDPE/50T50Z, and LLDPE/75T25Z.

### 2.4. Characterization 

The polymer nanocomposites had been characterized using multiple techniques. The morphology was observed using field emission scanning electron microscopy (FESEM, LEO GEMINI, Carl Zeiss, Oberkochen, Germany). The samples were gold-coated to avoid charging effect. The crystalline phases were analyzed using X-ray diffraction (XRD, X-Ray diffractometer D8 Advance BRUKER AXS GmbH, Karlsruhe, Germany). The degree of crystallinity (Xc) was calculated based on the following expression [36];

*X_c_* = (*I_c_*^110^ + *I_c_*^220^)/(*I_a_* + *I_c_*^110^ + *I_c_*^220^) (1)

where *I_a_* and *I_c_^hkl^* are the areas under the amorphous halo and the *hkl* reflections, respectively. The melting temperature (*Tm*) and the enthalpy of fusion (∆*H_f_*) were determined with a differential scanning calorimetry (DSC, Mettler Toledo, Kowloon, Hong Kong). Each nanocomposite with ~10–15 mg was weighed and scanned from room temperature until 200 °C at a heating rate of 20 °C/min in an inert nitrogen (N_2_) atmosphere (N_2_ flow rate, 50 mL/min). The relative crystallinity was determined from the enthalpy value (∆*H*); the parameter value for 100% crystalline LLDPE was 276 J/g [37]. The relative crystallinity was calculated based on the following equation [38]:

(2) $$\left(1-\lambda \right)\%=\frac{\Delta H_{f}}{\Delta H_{f100\%\;}^{{}^{\circ}}\cdot w}\times 100$$

*w* = weight fraction of the filler or matrix in blends, ∆*H_f_* = apparent enthalpy of melting of the filler or matrix, $\Delta H_{f100\%\;}^{{}^{\circ}}$ = extrapolated value of the enthalpy that corresponded to the melting of 100% crystalline sample (276 J/g for LLDPE). Next, the mechanical strength was examined using an Instron universal testing machine (Model TTC, Instron Corp., Buckinghamshire, UK). The metal ions released from the LLDPE nanocomposites were quantified using an inductively coupled plasma-optical emission spectrometer (ICP-OES Optima DV Perkin Elmer Inc., Branford, CT, USA). Each LLDPE nanocomposite film was (60 mm in diameter) immersed into a solution of 100 mL of deionized water, wherein 3 mL from the solution had been analyzed at 12, 24, 48, 72, and 96 h. The role of reactive species from LLDPE nanocomposites was determined by testing the photocatalytic activity of the nanocomposite films to degrade methylene blue (MB) aqueous solution. The film was immersed in 10 ppm of 40 mL of MB and kept in dark for 1 h to attain an equilibrium adsorption state. In order to determine the photodegradation efficiency under sunlight, a number of experiments were performed from 11:00 until 14:00. At fixed time interval, the LLDPE film was removed, while the solution was withdrawn and measured using a UV–vis spectrophotometer at 644 nm. The photocatalytic experiments were repeated in the presence of radical scavengers (5 mmol/L) to assess the impact of various reactive species (^•^OH, O_2_^•−^, h^+^) on the photocatalytic reaction. Methanol (MeOH), acetonitrile (ACN), and benzoquinone (BQ) were selected as scavengers for h^+^, ^•^OH radical, and superoxide anion radical O_2_^•−^, respectively.

### 2.5. Measurement of Antibacterial Ctivity

The antibacterial activity of LLDPE nanocomposites was determined using Gram-positive and Gram-negative strains. Standard ATCC (American Type Culture Collection) cultures, such as *S. aureus* ATCC 25923 and *E. coli* ATCC 25922, were employed in this study. The pathogens were grown aerobically in Luria-Bertani (LB) broth, (Merck, Darmstadt, Germany) for 24 h at 37 °C.

The antibacterial activity was assessed by adhering to the ASTM E2149-01 (ASTM Designation E 2149-01) standard. All glassware was sterilized before use. The bacterial culture was grown on a LB agar plate for 24 h. The grown culture was used to inoculate 50 mL of LB broth in a 100 mL Scott bottle. The culture was incubated at 37 °C with 115 rpm for 18–20 h. After that, the test cultures were diluted using sterilized buffer solution (pH 7.0) to reach a final concentration of (1.5–30) × 10^5^ cfu/mL. In order to evaluate the antibacterial activity, the test sample was added into a 250 mL conical flask filled with 50 mL of the working bacterial dilution. The conical flasks were shaken (115 rpm) for 1, 6, 12, and 24 h at 37 °C using a mechanical shaker under visible light (electricity fluorescent fixture T5 8W). After 1 h, 100 µL aliquot of appropriate dilution was aseptically pipetted to determine the bacterial concentration via standard plate counting method. The obtained value was converted into colony forming units per millimeter (cfu/mL) and had been considered as bacteria concentration at the initial contact time (t_0_). The bare LLDPE composite and the bacterial inoculum functioned as control. The colonies were counted and compared to those on the control plates to detect any change that might indicate inhibition of cell growth. The percentage of bacteria reduction (R%) was calculated using the following equation:

R% (cfu/mL) = [(B − A)/B] × 100,(3)

where R is antibacterial rate (%), B is the average number of cell colony of sample (cfu/sample) at t_0_ (1 h) contact time, and A is the average number of colonies of treated sample (cfu/sample) within specified contact time [39].

## 3. Results and Discussion

### 3.1. Surface Morphology of LLDPE Nanocomposites

**Figure 1:**
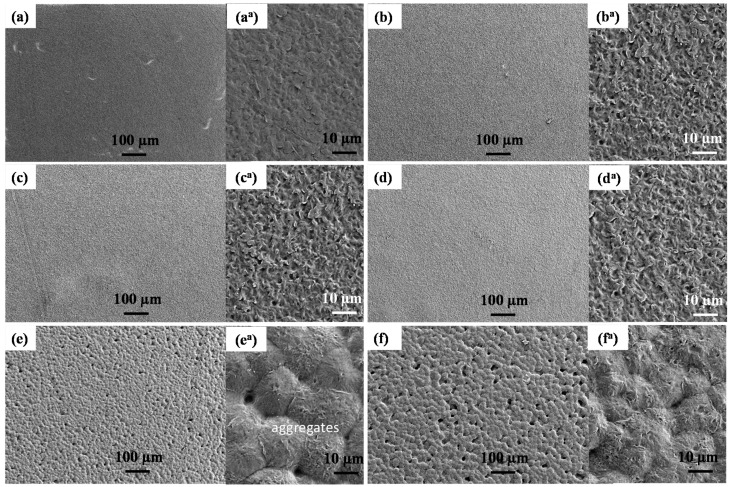
FESEM (magnification: 100×) images of surface morphology of (**a**) bare linear low-density polyethylene (LLDPE), (**b**) LLDPE/100T, (**c**) LLDPE/75T25Z, (**d**) LLDPE/50T50Z, (**e**) LLDPE/25T75Z, and (**f**) LLDPE/100Z. Illustrations on the right refer to high magnification (1000×) FESEM images (**a^a^**–**f^a^**) of the corresponding LLDPE nanocomposites.

FESEM images of bare and LLDPE nanocomposites are illustrated in Figure 1. The surface morphology of bare LLDPE (Figure 1a) and nanocomposites with high TiO_2_ content (100T, 50T50Z, and 75T25Z) (Figure 1b–d) displayed relatively similar smooth surfaces. The samples seemed to have a dense texture. On the contrary, LDPE nanocomposites with high ZnO content (100Z, 25T75Z) were rather rough with distinct voids (Figure 1e,f). This characteristic is important to increase the production and migration rates of Zn^2+^ ions within the LLDPE matrix (refer to ICP analysis in Section 3.7), thus enhancing the antimicrobial activity. The nanoparticles were homogeneously distributed on the surface (inset Figure 1b^a^–f^a^ and supporting Figure S1) as evident by small white dots and aggregation of several nanoparticles. The EDX analysis also revealed the presence of Zn, Ti, C and O (Figure S2). The presence of nanoparticles on the surface is expected to enhance the antimicrobial activity rapidly. 

### 3.2. Crystal Analysis of LLDPE Nanocomposites

**Figure 2:**
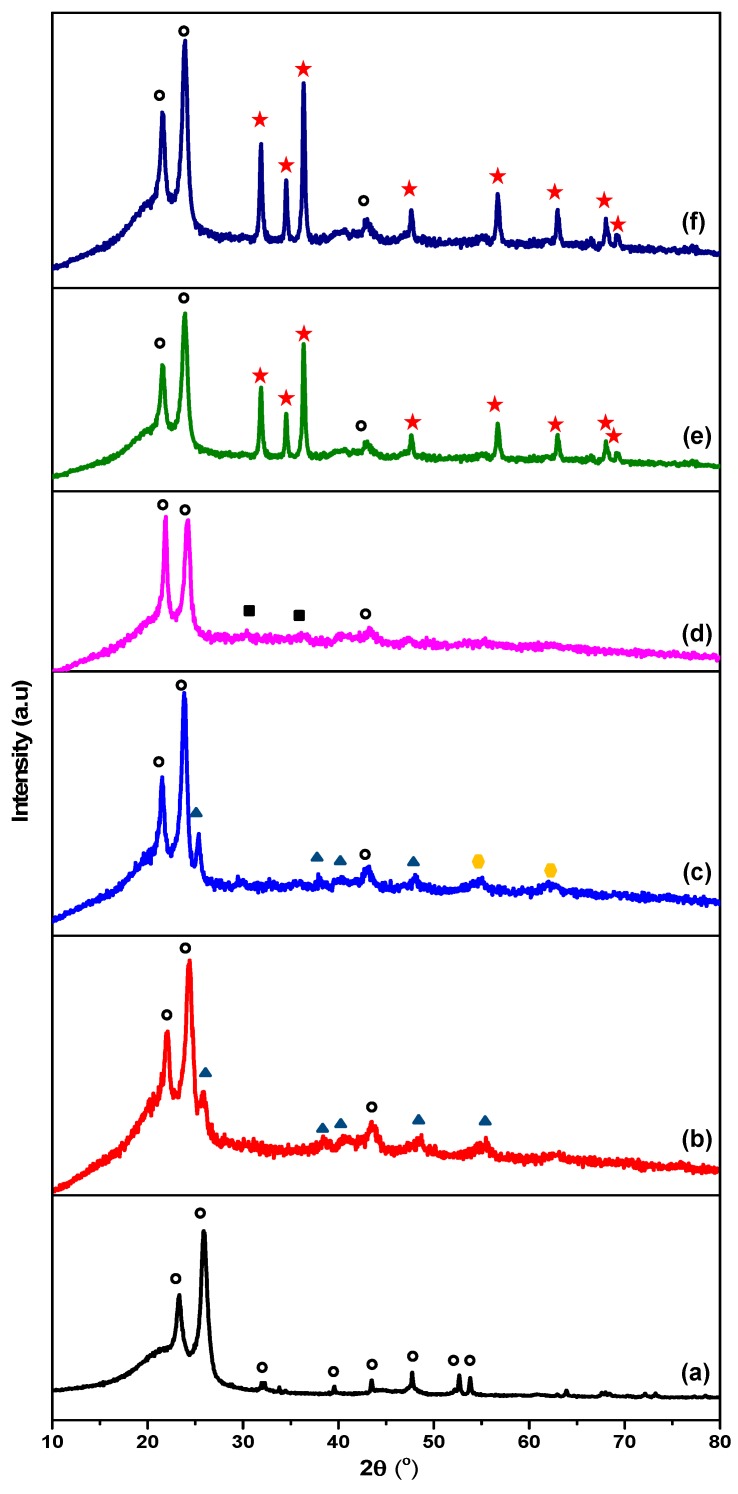
XRD patterns of (a) bare LLDPE, (b) LLDPE/100T, (c) LLDPE/75T25Z, (d) LLDPE/50T50Z, (e) LLDPE/25T75Z, and (f) 100Z. [○: LLDPE, ▲: Anatase, ⬣: ZnTiO_3_, ■: Zn_2_Ti_3_O_8_, ★: Zincite (ZnO)].

**Figure 3:**
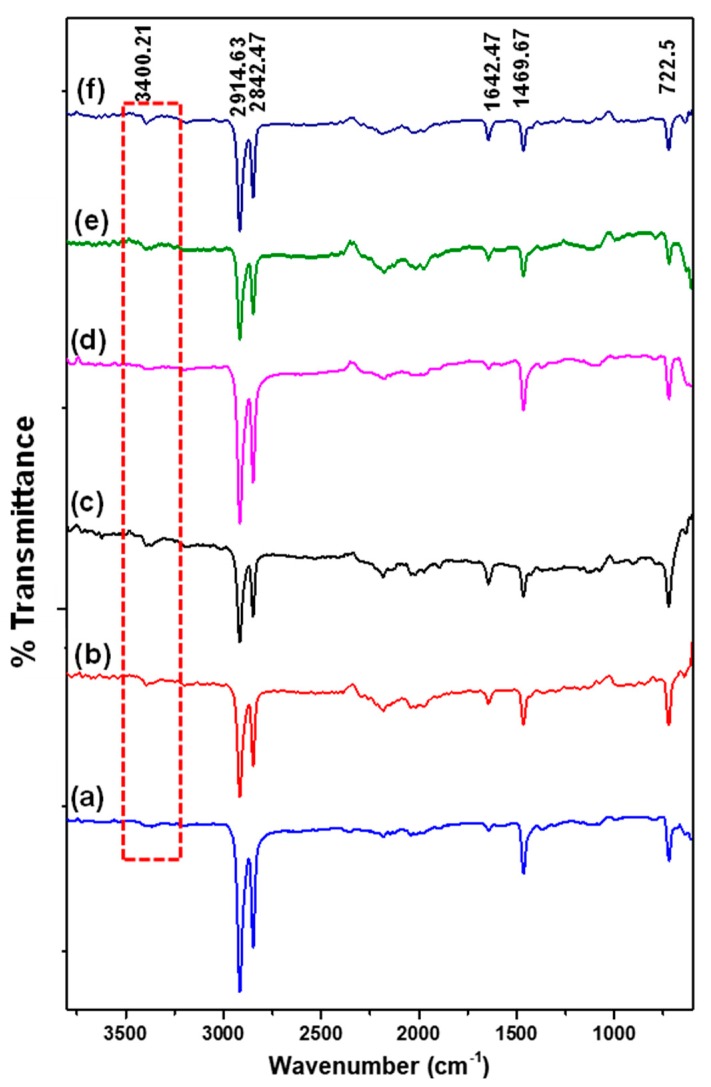
FT-IR spectra of samples (a) bare LLDPE, (b) LLDPE/100T, (c) LLDPE/75T25Z, (d) LLDPE/50T50Z, (e) LLDPE/25T75Z, and (f) LLDPE/100Z.

Figure 2 portrays the XRD patterns of bare and LLDPE/metal oxide nanocomposites. As for bare LLDPE (Figure 2a), two distinct peaks were observed at 22.5° and 24.5°, which represented (110) and (220) reflections of orthorhombic LLDPE [40]. Meanwhile, for LLDPE/100T (Figure 2b), an additional anatase peak of TiO_2_ was noted at 2θ = 25.5° (JCPDS21-1272), indicating that most of the TiO_2_ nanoparticles were embedded in the polymer matrix, whereas some remained on the surface [41]. LLDPE/75T25Z seemed to share similar characteristics with LLDPE/100T as three distinctive diffraction peaks were clearly noticed (Figure 2c). Only characteristic peaks of LLDPE were recorded for LLDPE/50T50Z (Figure 2d), which can be ascribed to the existence of cubic Zn_2_Ti_3_O_8_ with low-degree crystallinity [Supporting Table S1]. The characteristic peaks of LLDPE (22.5 and 24.5°) and the three distinctive diffraction peaks of hexagonal zincite (2θ = 31.7, 34.4, and 36.2° for (100), (002), and (101) planes, respectively) were observed for LLDPE/25T75Z and LLDPE/100Z composites [42]. Based on the outcomes derived from XRD, the main characteristic peaks of LLDPE appeared to have been retained in all samples, indicating that the incorporation of coupled TiO_2_/ZnO nanoparticles did not significantly alter the general structure of LLDPE matrix, while the interaction between oxide nanoparticles and LLDPE involved a physical process, as verified via FT-IR (Figure 3). However, the crystallinity index of LLDPE decreased upon reaction with highly crystalline metal oxide (Table 1). It is testified that the incorporation of 100T, 75T25Z, 25T75Z, and 100Z metal oxides has appreciable influence on the crystallinity of LLDPE, in comparison to oxide with low-degree crystallinity (50T50Z). As noted, highly crystalline metal oxides reduced the degree of crystallinity of LLDPE and enhanced the water uptake through polymer matrix [43]. Hence, the antimicrobial properties of LLDPE nanocomposites were expected to enhance. 

### 3.3. FT-IR

FT-IR is one of the most widely used tools for polymer characterization due to its sensitivity towards molecular environment, as well as chain conformation and morphology, particularly concerning the detection of energy levels transition in macromolecules [44,45]. Figure 3 shows the FT-IR spectrum of bare and LLDPE/metal oxide nanocomposites. Bare LLDPE (Figure 3a) revealed characteristic peaks at 2914.63 and 2842.47 cm^−1^, indicating C-H stretching vibrations of CH_2_ and CH groups, respectively [46]. Another band recorded at 1642.67 cm^−1^ could be associated to CH_3_ bonding deformation. The peaks at 1469.67 and 722.5 cm^−1^ are attributable to CH_2_ bending deformation and rocking deformation, respectively. The FT-IR spectrum of LLDPE nanocomposites seemed to be similar to that of bare LLDPE, except for a shallow broad peak of O–H at 3400.21 cm^−1^ (red-dashed frame), which refers to a characteristic band for hydroxyl group at the surface of the metal oxides [47]. The presence of LLDPE characteristics in the nanocomposites were noted with slight reduction in terms of intensity, hence signifying that the chemical structures have been preserved without the formation of appreciable chemical bonds between LLDPE and nanoparticles. The peak intensity for LLDPE/50T50Z at 2914.63 and 2842.47 cm^−1^ was more intense, when compared to other LLDPE nanocomposites, which can be attributed to the intense stretching vibration of LLDPE matrix.

### 3.4. Mechanical Properties

Table 2 presents the findings obtained for Young modulus’ tensile strength (TS) and percentage of elongation of bare and LLDPE nanocomposites. Bare LLDPE exhibited lower TS and Young modulus, but higher percentage of elongation, in comparison to those of nanocomposites. Upon integration of nanoparticles, TS and Young modulus seemed to increase, but reduction was recorded for the percentage of elongation, which is in agreement with other investigations [48,49]. The nanoparticles in LLDPE matrix displayed toughening and reinforcing effects that restricted the molecular motion of LLDPE polymer chains, thus enhancing the capability of LLDPE to withstand greater loads [41]. When inorganic nanoparticles were amalgamated into the LLDPE matrix, the percentage of elongation dropped drastically mainly because the nanoparticles hindered dislocation motion, thus decreasing the stress at break. On the contrary, bare LLDPE showcased sufficient space and time to orientate, especially when force was applied to the polymer chains. In order to evaluate the biocidal activity, samples that possessed more strength, moderate Young modulus, and elongation had been essential. This is because LLDPE matrix with more amorphous region may enhance the release of metal ion during water uptake.

### 3.5. Thermal Analysis

Table 3 displays the parameters of thermal analysis (crystallization temperatures (*Tc*), melting temperatures (*Tm*), apparent enthalpy (H′), melting enthalpy (∆H_o_), and degree of crystallinity (% X_c_)) for bare and LLDPE nanocomposites. A slight increment was noted in *Tc* and *Tm* for LLDPE nanocomposites, when compared to that for bare LLDPE. This can be ascribed to the high thermal stability of the nanocomposites from the physical hindrance of nanoparticles to the motion of LLDPE molecular chain. Nevertheless, the H′ value for all LLDPE nanocomposites, except for LLDPE/50T50Z, seemed to have slumped. In fact, a similar trend was noted for %X_c_. The crystallinity of LLDPE was influenced by the spherulitic growth. Meanwhile, in LLDPE nanocomposites, the growth of spherulites was hindered by the presence of crystalline nanoparticles between the LLDPE polymeric chains; hence, a retard in the nucleation of LLDPE crystal [50]. This is because crystalline inorganic nanoparticles do not co-crystallize with LLDPE, and thus inhibit LLDPE crystallization. Therefore, the degree of crystallinity for LLDPE nanocomposites was relatively lower than that for bare LLDPE, hence contributing to more amorphous region for better bactericidal effect. The %X_c_ value of LLDPE/50T50Z was similar to that of LLDPE. This is attributable to the amorphous nature of nanoparticles, in which LLDPE crystallization did not decrease to a greater extend as that in LLDPE nanocomposites with crystalline nanoparticles.

### 3.6. Relative Production of Reactive Species (Hydroxyl Radicals, Superoxide Anion and Holes)

**Figure 4:**
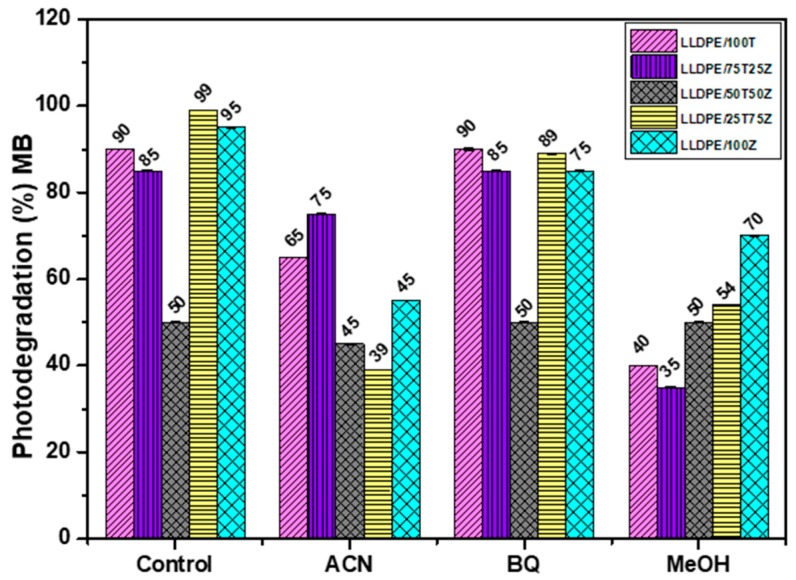
Influence of radical scavengers on photodegradation of MB over LLDPE/100T, LLDPE/75T25Z, LLDPE/50T50Z, LLDPE/25T75Z, and LLDPE/100Z, under sunlight: Control (LLDPE nanocomposite with MB alone), BQ as scavenger for O_2_^•^¯radical, MeOH as scavenger for h^+^ and ACN scavenger for ^•^OH radical.

The photo-activated surface properties of LLDPE nanocomposites were considered for the ability of light-activated disinfection in TiO_2_/ZnO nanoparticles. Hence, the photocatalytic activity of LLDPE nanocomposites on degradation of organic dye (methylene blue, MB) was examined in the presence of radical scavengers to quantify the migration of reactive species (^•^OH radical, superoxide anion, and h^+^). Acetonitrile (ACN), benzoquinone (BQ), and methanol (MeOH) were selected as scavenger for ^•^OH, O_2_^•−^, and h^+^, respectively. Figure 4 illustrates the percentage of photo-degradation of MB using LLDPE nanocomposites in the presence of radical scavengers. As observed, the photodegradation efficiency displayed by LLDPE composites with 100T, 75T25Z, 50T50Z, 25T75Z, and 100Z were 90%, 85%, 50%, 99%, and 95%, respectively, in the absence of radical scavengers; which revealed that 25T75Z had the highest release of reactive species. Upon the introduction of ACN, the photodegradation efficiency of 100Z and 25T75Z decelerated drastically by 50% and 60%, respectively (Table 4). On the other hand, the efficiency of 100T, 75T25Z, and 50T50Z decreased marginally by 15%, 10%, and 5%, respectively. The outcomes reveal that ^•^OH is the main reactive species in LLDPE nanocomposites. When BQ (a scavenger for O_2_^•−^ radical) was added to the reaction, the photodegradation of MB for 100T, 75T25Z, and 50T50Z remained constant, indicating zero participation of O_2_^•−^ in those samples rich with crystalline TiO_2_ and partially crystalline Zn_2_Ti_3_O_8_. Nonetheless, the efficiency of LLDPE with 100Z and 25T75Z changed by 20% and 10%, signifying that 100Z induced greater O_2_^•^¯ from film, when compared to 25T75Z. With the addition of MeOH, the degradation of both samples (100T and 75T25Z) reduced by 50%, which demonstrated the major contribution of h^+^ in photocatalytic reaction. Besides, the reduction in 25T75Z and 100Z by 40% and 25%, respectively, there was higher participation of h^+^ in the photocatalytic reaction of 25T75Z, when compared to that in 100Z. Overall, the findings appear to confirm the photocatalytic activity for bacteriostatic effect of LLDPE nanocomposites rich with zincite phase (100ZnO and 25T75Z), which were primarily driven by three reactive species in a sequence of ^•^OH > O_2_^•−^ > h^+^ and ^•^OH > h^+^ > O_2_^•−^, respectively. As for the photocatalytic activity of LLDPE nanocomposites rich with TiO_2_ (100T and 75T25Z), two reactive species contributed in a sequence of h^+^ > ^•^OH, whereas in 50Z50T, minor participation of ^•^OH was involved.

### 3.7. Zinc Ion Release

**Figure 5:**
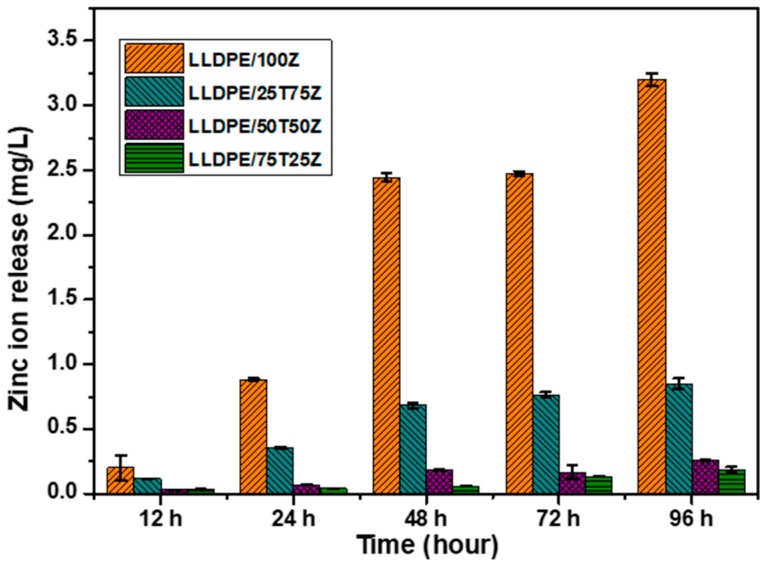
Zn^2+^ ion concentration in deionized water after immersion periods of 12–96 h using LLDPE nanocomposites.

The release of metal ion from LLDPE nanocomposites also has an integral role in determining the antibacterial properties of LLDPE nanocomposites. In this study, the release of zinc ions (Zn^2+^) was evaluated by immersing LLDPE nanocomposites in water for 12 h until 96 h. After the immersion period, the water was analyzed to determine the dissolved Zn^2+^ ions, as shown in Figure 5. For the entire LLDPE nanocomposites, the release of Zn^2+^ increased with time, which is attributed to the disintegration of the existing intermolecular hydrogen bonding between LLDPE and water molecules. Water diffusion has an impact on the plasticity of LLDPE matrix and permits more favorable conditions for the release of Zn^2+^ ions. The outputs demonstrate that the rate of Zn^2+^ ion release differed for each LLDPE nanocomposite. The release of Zn^2+^ ions was significantly higher for LLDPE/100Z, and followed by LLDPE/25T75Z. The other LLDPE nanocomposites, however, did not exhibit appreciable release within the fixed time. The high rate of Zn^2+^ ion release showcased in LLDPE/100Z is ascribed to enhanced water diffusion characteristics and the amorphous nature of LLDPE nanocomposite. The voids in LLDPE/100Z (Figure 1f) may have accelerated the entry of water molecules and the migration of zinc ions. In light of this, LLDPE/25T75Z with steady increment could stand a better chance for long-term application, in comparison to LLDPE/100Z. This is yet to be proven and the related research is ongoing. Overall, the outcomes support low-degree polymer matrix, presence of ZnO phase, nanoparticles with hydrophilic nature, and void-controlled ion release.

### 3.8. Antibacterial Properties

**Figure 6:**
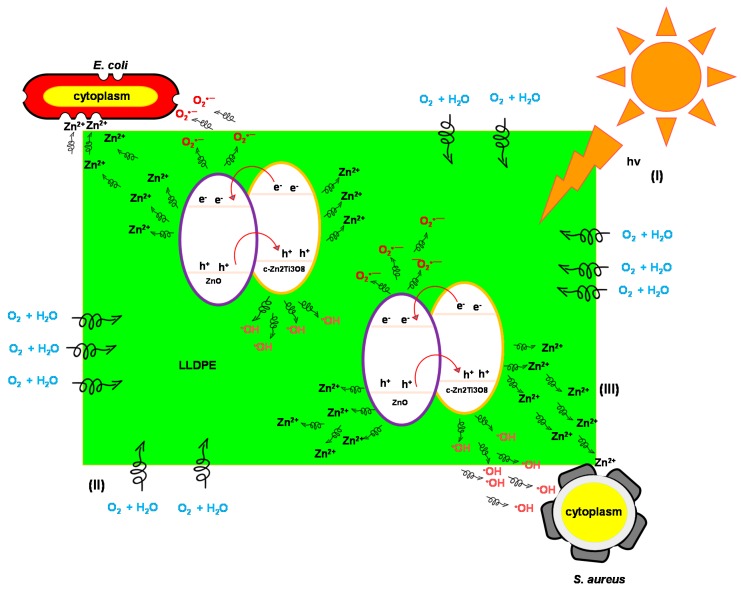
Antimicrobial mechanism of LLDPE/25T75Z nanocomposites against *S. aureus* and *E. coli.*

Gram-positive (*S. aureus*) and Gram-negative (*E. coli*) were tested by adhering to the ASTM E 2149 test method. The results (Table 5) show that all LLDPE nanocomposites displayed remarkable efficacy against *S. aureus*. Nonetheless, only LLDPE nanocomposites with ZnO exhibited antibacterial activity against *E. coli* (Table 6). Figure 6 illustrates the bacterial reduction percentages for both *S. aureus* and *E. coli* with LLDPE nanocomposites immersed for 24 h in LB broth. LLDPE/100T and LLDPE/75T25Z inactivated *S. aureus* by approximately 77% and 95 %, respectively. Nevertheless, both were inactive against *E. coli*. LLDPE/50T50Z, LLDPE/25T75Z, and LLDPE/100Z showed exceptional bacteriostatic effect on *S. aureus* and *E. coli*. These outcomes signify that LLDPE nanocomposites rich in ZnO (LLDPE/25T75Z and LLDPE/100Z) nanoparticles could effectively destroy both pathogens. 

Based on the scavenger and ion release analyses, two primary mechanisms were determined for the bacteriostatic effect: (1) inclusion of oxidative stress through the interaction of reactive species (^•^OH radicals and h^+^) with proteins, DNA, and lipids; and (2) release of Zn^2+^ ions that may be responsible for antibacterial activity by binding to the cell membrane. The antibacterial effect of LLDPE/100T on *S. aureus* seemed to be mainly driven by ^•^OH radicals and h^+^. Obviously, these outputs explain the superior bacteriostatic effect of LLDPE/25T75Z and LLDPE/100Z upon *S. aureus*, which generated more ^•^OH radicals and h^+^, when compared to LLDPE/100T [51]. The presence of voids and relatively low crystallinity nature of LLDPE/25T75Z and LLDPE/100Z expedited water uptake through polymer matrix, which promoted the release of reactive species, when compared to that in compact LLDPE/100T.

LLDPE/100T did not display any impact upon *E. coli*. This suggests that Zn^2+^ may be the sole responsible species to destroy *E. coli*. Based on the XRD analysis, LLDPE/25T75Z and LLDPE/100Z revealed high content of ZnO phase that was hydrophilic in nature, so as to enhance water penetration via polymer matrix and consequently generate hydrated Zn^2+^ ions. *E. coli* has a negative surface charge membrane with a layer of cytoplasm and multilayers of peptidoglycan polymer [26]. Therefore, the positively-charged Zn^2+^ ion species can easily penetrate into the surface of *E. coli* cell wall membrane. The released Zn^2+^ ions from the polymer matrix would accumulate in the cell due to electrostatic forces. This disrupts normal cellular activities, thus resulting in membrane disorder. Zn^2+^ ions also have strong chemical affinity towards thiol groups of macro-biomolecules, which could inhibit protein synthesis, nucleic acid synthesis, and metabolic pathways. Interruption of trans-membrane electron transportation by Zn^2+^ ions also can hamper microbial growth [26]. 

**Figure 7:**
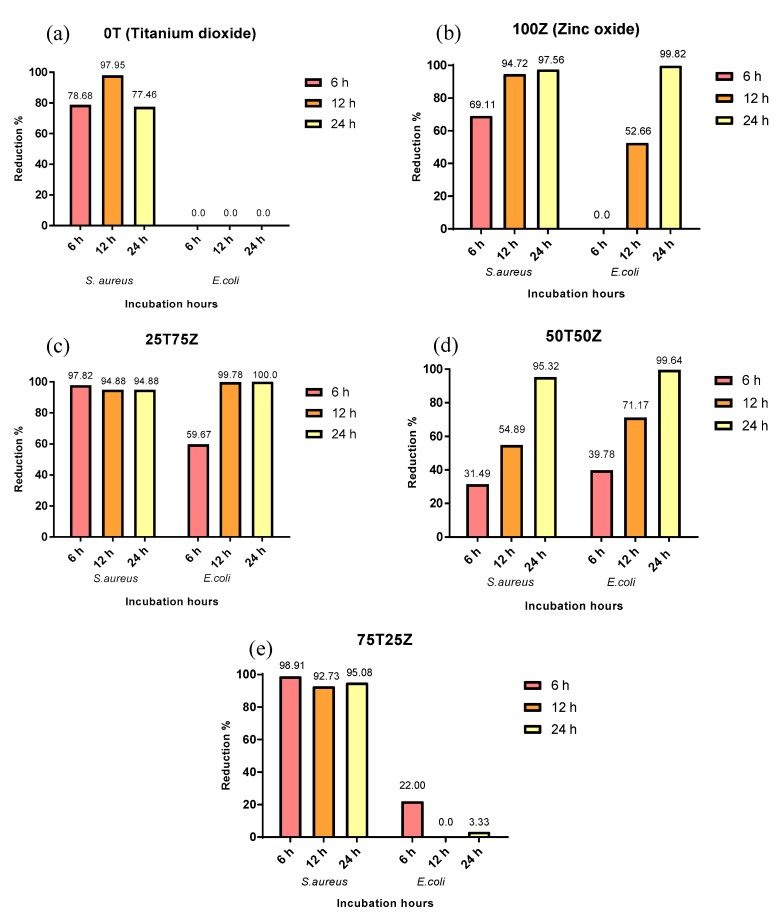
Antibacterial efficiency of LLDPE nanocomposites with different metal oxide ratios (**a**) LLDPE/100T, (**b**) LLDPE/100Z, (**c**) LLDPE/25T75Z, (**d**) LLDPE/50T50Z, and (**e**) LLDPE/75T25Z against *S. aureus* and *E. coli*.

In this study, TiO_2_/ZnO nanocomposite (LLDPE/25T75Z) showcased better antibacterial performance against *E. coli* and *S. aureus*, in comparison to TiO_2_ or ZnO nanocomposite. The discrepancy between the inactivation capabilities of these samples at 6 h and 12 h can be explained by the significant fact that TiO_2_/ZnO retained effective charge separation due to the formation of heterojunction between ZnO and c-Zn_2_Ti_3_O_8_ (Supporting Figure S2), which aided the transfer of electron from conduction band (CB) of ZnO to CB of c-Zn_2_Ti_3_O_8_ and h^+^ from the valence band (VB) of c-Zn_2_Ti_3_O_8_ to that of ZnO [27]. As a result, more e^−^ and h^+^ were made available for ^•^OH radical formation, and were hence released from LLDPE nanocomposite to inactivate the pathogens (Figure 4). Figure 7 portrays that the release of Zn^2+^ ions appeared to be greater in LLDPE nanocomposites with high ZnO weight ratio. Nevertheless, LLDPE/25T75Z displayed complete destruction of *E. coli*, although the release of Zn^2+^ ions had been lower than that of 100Z. This is attributable to the minor effect of O_2_^•−^ radicals generated by LLDPE/25T75Z (Figure 4), which led to H_2_O_2_ generation. Upon contact with *E. coli* suspension, the h^+^ would split H_2_O molecules into OH¯ and H^+^. Next, the dissolved oxygen molecules are transformed to O_2_^•−^ radicals, which in turn, react with H^+^ to generate (HO_2_^•^) radicals, which, upon subsequent collision with electrons, produce hydrogen peroxide anions (HO_2_¯). After that, they then react with H^+^ to produce H_2_O_2_ molecules [52]. Since ^•^OH and O_2_^•−^ are negatively-charged particles, they cannot easily penetrate into the cell membrane and must remain in direct contact with the outer surface of the bacteria. On the contrary, H_2_O_2_ molecules can penetrate easily into the cell. These radicals will attack the polyunsaturated fatty acids in the cell membrane and cause lipid peroxidation, which in turn, cause cell death. Therefore, it can be concluded that the higher efficacy displayed by LLDPE/25T75Z can be attributed to the release of Zn^2+^ and the production of O_2_^•−^ radicals. 

LLDPE/50T50Z appeared to be less effective below 24 h, and the reason is probably due to the dominant c-Zn_2_Ti_3_O_8_ phase with low-degree crystallinity, which resulted in low photocatalytic reaction. Besides, the C-index of this sample was relatively higher, suggesting slow water uptake through the polymer matrix for ion or reactive species release. Figure 6 presents the findings for antibacterial mechanism of LLDPE nanocomposites against *S. aureus* and *E. coli*. Under sunlight illumination, a pair of negatively-charged electrons and a positively-charged electron hole had been created, as illustrated in reaction pathway (I). Upon contact with O_2_ and H_2_O when they reached the surface, the O_2_^•−^ and ^•^OH radicals were generated, respectively (Equations (4) and (5)). These radicals are extremely reactive when in contact with bacteria. The initial oxidative damage occurs on the cell wall where LLDPE/25T75Z photocatalytic surface makes the first contact with the intact cell. With increment in contact time, the diffusion of H_2_O is also increased (pathway (II)), thus allowing O_2_^•^¯ to react with H^+^ so as to generate HO_2_^•^ radicals and subsequently produce HO_2_^−^ (Equation (6)). These, in solution, can react with H^+^ to form H_2_O_2_ (Equation (7)). The generated H_2_O_2_ is diffused into the inner membrane and causes cell membrane damage. Damage inflicted upon the cell membrane directly leads to leakage of minerals, proteins, and genetic materials, thus causing cell death. Concurrently, the leaching of Zn^2+^ from LLDPE/25T75Z into the growth media that occurs in pathway (III) promotes cell death. Therefore, the toxicological mechanism of Zn^2+^ plays an important role in rupturing the cell wall by degrading lipoprotein C- and N-terminals, which is the cause for the destruction of the outer membrane structure.

O_2_ + e^−^ = O_2_^•−^ (4)

H_2_O + h^+^ = ^•^OH + H^+^ (5)

O_2_^•−^ + H^+^ = HO_2_^•^ (6)

HO_2_^•^ + H^+^ + e^−^ = H_2_O_2_ (7)

## 4. Conclusions

LLDPE/metal oxide nanocomposites were synthesized through wet mixing method. The crystalline phases, the existence of metal oxide at the polymer matrix (bulk and/or surface), related voids, the degree of LLDPE crystallinity, the hydrophilic nature, the reactive species release (^•^OH, h^+^, and Zn^2+^ ion release), and the antimicrobial efficiency were studied in detail. This study discovered that LLDPE nanocomposite with high ZnO content displayed remarkable antibacterial efficiency against *E. coli* and *S. aureus*. The reactive species release (^•^OH, h^+^, and Zn^2+^ ion release) was enhanced by low crystalline LLDPE matrix with voids. Inactivation of *E. coli* was mainly driven by Zn^2+^ ion release, whereas ^•^OH contributed to the hampering of *S. aureus*. The formation of heterojunction also appears to be essential in charge carrier separation to inactivate the pathogens at a rapid rate.

## Figures and Tables

**Table 1:** Position of crystalline peaks and crystal indices for bare LLDPE and LLDPE nanocomposites with different ratios of TiO_2_/ZnO nanoparticles.

Samples	2θ (°) (110)	*d*-Spacing (nm)	2θ (°) (220)	*d*-Spacing (nm)	*C*-Index
LLDPE	21.75	4.08	24.14	3.70	0.64
LLDPE/100T	21.88	4.10	24.88	3.72	0.49
LLDPE/75T25Z	21.80	4.10	24.67	3.71	0.45
LLDPE/50T50Z	21.78	4.11	24.77	3.72	0.56
LLDPE/25T75Z	21.83	4.08	24.81	3.71	0.36
LLDPE/100Z	21.81	4.11	24.90	3.71	0.43

**Table 2:** Tensile strength, Young modulus, and percentage of elongation recorded for bare and LLDPE nanocomposites with different TiO_2_/ZnO ratios.

Films	Tensile Strength (MPa)	Young Modulus (MPa)	Percent Elongation (%)
LLDPE	5.99 ± 0.14	91.93 ± 1.77	62.80 ± 18.67
LLDPE/100T	8.89 ± 0.61	122.66 ± 8.17	41.50 ± 7.80
LLDPE/75T25Z	9.21 ± 0.20	157.51 ± 4.48	24.73 ± 7.62
LLDPE/50T50Z	8.45 ± 0.42	164.03 ± 7.76	20.40 ± 1.37
LLDPE/25T75Z	8.16 ± 1.10	131.32 ± 19.46	35.20 ± 9.84
LLDPE/100Z	9.55 ± 0.56	181.54 ± 8.12	15.97 ± 3.23

**Table 3:** *Tc*, *Tm*, H′, ∆H_o_, and percent crystallinity, X_c_ of bare LLDPE and LLDPE nanocomposites with different TiO_2_/ZnO ratio.

Sample	*Tc* (^o^C)	*Tm* (^o^C)	Apparent Enthalpy H′ (J/g) = Enthalpy of Fusion, H_m_ − Enthalpy of Crystallisation, H_c_	Enthalpy of Melting of 100% Crystalline LLDPE, ∆H_o_ (J/g)	Percent Crystallinity, X_c_ (%)
LLDPE	36.5	106.7	52.53	276	19.03
LLDPE/100T	36.4	108.2	37.94	276	13.74
LLDPE/75T25Z	39.7	107.5	33.21	276	12.26
LLDPE/50T50Z	38.9	107.6	50.42	276	18.24
LLDPE/25T75Z	38.8	108.0	44.45	276	15.59
LLDPE/100Z	38.9	107.6	41.97	276	15.20

**Table 4:** ^•^OH radical scavenging activity, O_2_^•−^radical scavenging activity, and h^+^ scavenging activity of LLDPE nanocomposites.

Sample	^•^OH Radical Scavenging Activity (%)	O_2_^•−^ Radical Scavenging Activity (%)	h^+^ Scavenging Activity (%)
LLDPE/100T	15	-	50
LLDPE/75T25Z	10	-	50
LLDPE/50T50Z	5	-	-
LLDPE/25T75Z	60	10	40
LLDPE/100Z	50	20	25

**Table 5:** A summary of antibacterial activity of TiO_2_/ZnO-LLDPE nanocomposites against *S. aureus* (cfu/mL expressed as mean ± standard deviation and percentage for bacterial reduction).

Time (h)	Samples									
	Inoculum (cfu/mL)					Bacterial Reduction (%)				
	100T	100Z	25T75Z	50T50Z	75T25Z	100T	100Z	25T75Z	50T50Z	75T25Z
1	(2.43 ± 0.39) × 10^4^	(2.46 ± 0.47) × 10^4^	(1.70 ± 0.04) × 10^4^	(2.35 ± 0.34) × 10^4^	(1.83 ± 0.10) × 10^4^	-	-	-	-	-
6	(0.52 ± 0.10) × 10^4^	(0.76 ± 0.05) × 10^4^	(0.37 ± 0.02) × 10^3^	(1.61 ± 0.01) × 10^4^	(0.20 ± 0.10) × 10^3^	78.68	69.11	97.82	31.49	98.91
12	(0.53 ± 0.06) × 103	(0.13 ± 0.02) × 10^4^	(0.87 ± 0.06) × 10^3^	(1.06 ± 0.03) × 10^4^	(1.13 ± 0.76) × 10^3^	97.95	94.72	94.88	54.89	92.73
24	(0.55 ± 0.24) × 10^4^	(0.6 ± 0.17) × 10^3^	(0.87 ± 0.21) × 10^3^	(0.11 ± 0.01) × 10^4^	(0.90 ± 0.44) × 10^3^	77.46	97.56	94.88	95.32	95.08

**Table 6:** A summary of antibacterial activity of TiO_2_/ZnO-LLDPE nanocomposites against *E. coli* (cfu/mL expressed as mean ± standard deviation and percentage for bacterial reduction).

Time (h)	Samples									
	Inoculum (cfu/mL)					Bacterial Reduction (%)				
	100T	100Z	25T75Z	50T50Z	75T25Z	100T	100Z	25T75Z	50T50Z	75T25Z
1	(3.0 ± 0.0) × 10^4^	(1.88 ± 0.30) × 10^4^	(3.0 ± 0.0) × 10^4^	(2.74 ± 0.28) × 10^4^	(3.0 ± 0.0) × 10^4^	-	-	-	-	-
6	(3.0 ± 0.0) × 10^4^	(3.0 ± 0.0) × 10^4^	(1.21 ± 0.09) × 10^4^	(1.65 ± 0.38) × 10^4^	(2.34 ± 1.10) × 10^4^	0	0	59.67	39.78	22.0
12	(3.0 ± 0.0) × 10^4^	(0.89 ± 0.15) × 10^4^	(0.66 ± 1.15) × 10^2^	(0.79 ± 0.20) × 10^4^	(3.0 ± 0.0) × 10^4^	0	52.66	99.78	71.17	0
24	(3.0 ± 0.0) × 10^4^	(0.33 ± 0.57) × 10^2^	-	(0.10 ± 0.10) × 10^3^	(2.90 ± 0.18) × 10^4^	0	99.82	100.0	99.64	3.33

## Supplementary Materials

The following are available online at http://www.mdpi.com/2073-4360/10/8/878/s1, Figure S1: FESEM (magnification: 1000×) images of surface morphology of (a) bare LLDPE, (b) LLDPE/100T, (c) LLDPE/75T25Z, (d) LLDPE/50T50Z, (e) LLDPE/25T75Z and (f) LLDPE/100Z; Figure S2: FESEM image and EDX spectrum of LLDPE/75T25Z; Figure S3: FESEM images of (a) 100T, (b) 100Z and (c) 25T75Z; Table S1: Phase present in 100T, 75T25Z, 50T50Z, 25T75Z and 100Z nanoparticles as obtained in XRD analysis; Table S2: At% of Zn an Ti of the corresponding metal oxide.
