# Supplementary material for: Bacteriostatic Activity of LLDPE Nanocomposite Embedded with Sol–Gel Synthesized TiO2/ZnO Coupled Oxides at Various Ratios
Source: Polymers (Basel). 2018 Aug 6;10(8):878. doi: 10.3390/polym10080878 (PMC6403739; doi:10.3390/polym10080878)
Supplement: Supplementary file 1 [file polymers-10-00878-s001.pdf]

Supporting file S1

Figure S1

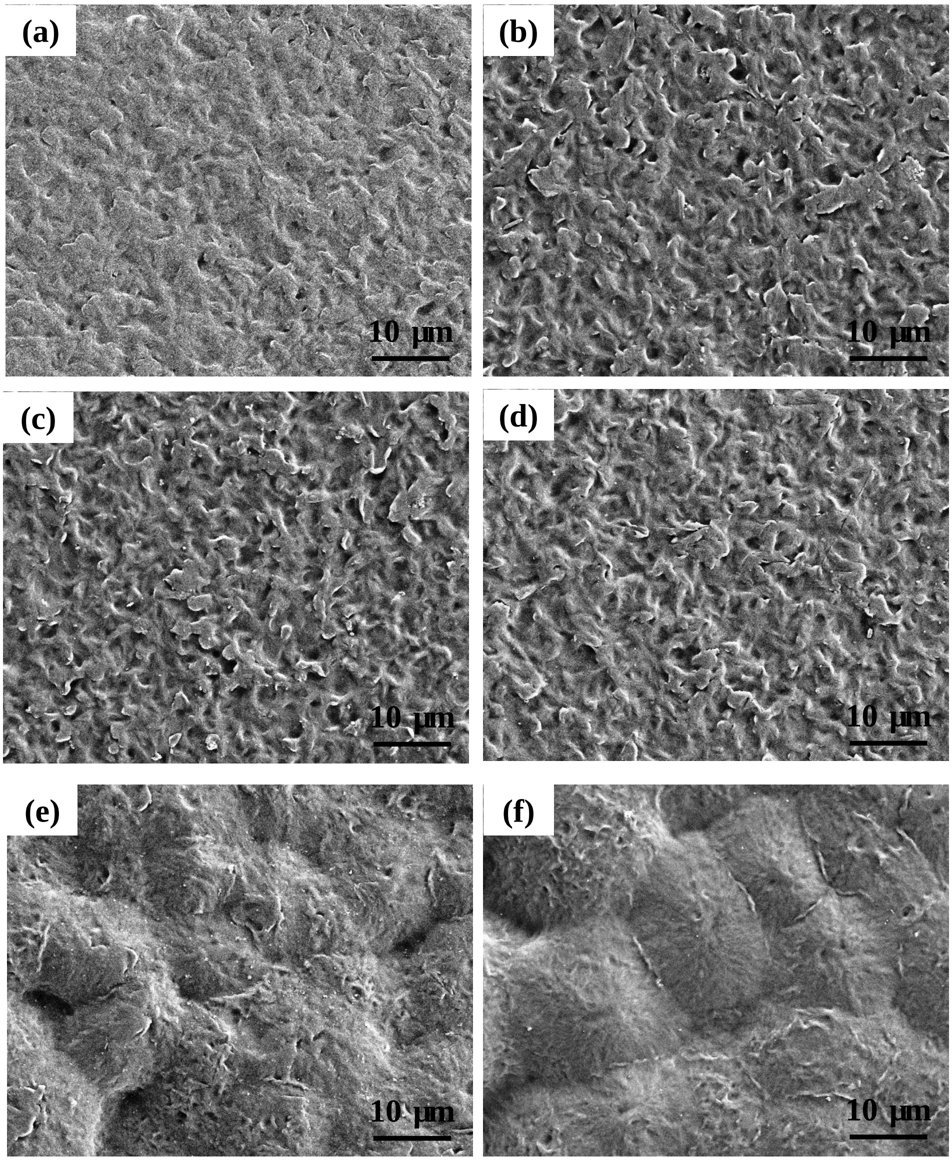


**Figure S1.** FESEM (magnification: 1000 x) images of surface morphology of (a) bare LLDPE, (b) LLDPE/100T, (c) LLDPE/75T25Z, (d) LLDPE/50T50Z, (e) LLDPE/25T75Z and (f) LLDPE/100Z.

Figure S2

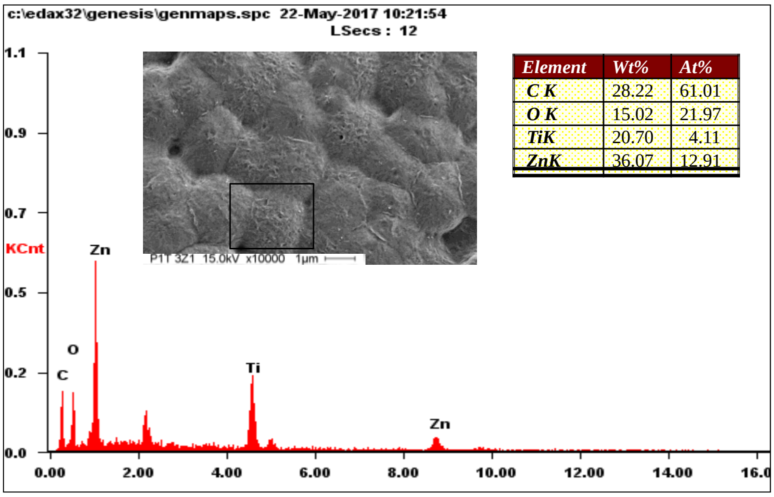


**Figure S2.** FESEM image and EDX spectrum of LLDPE/75T25Z

Figure S3

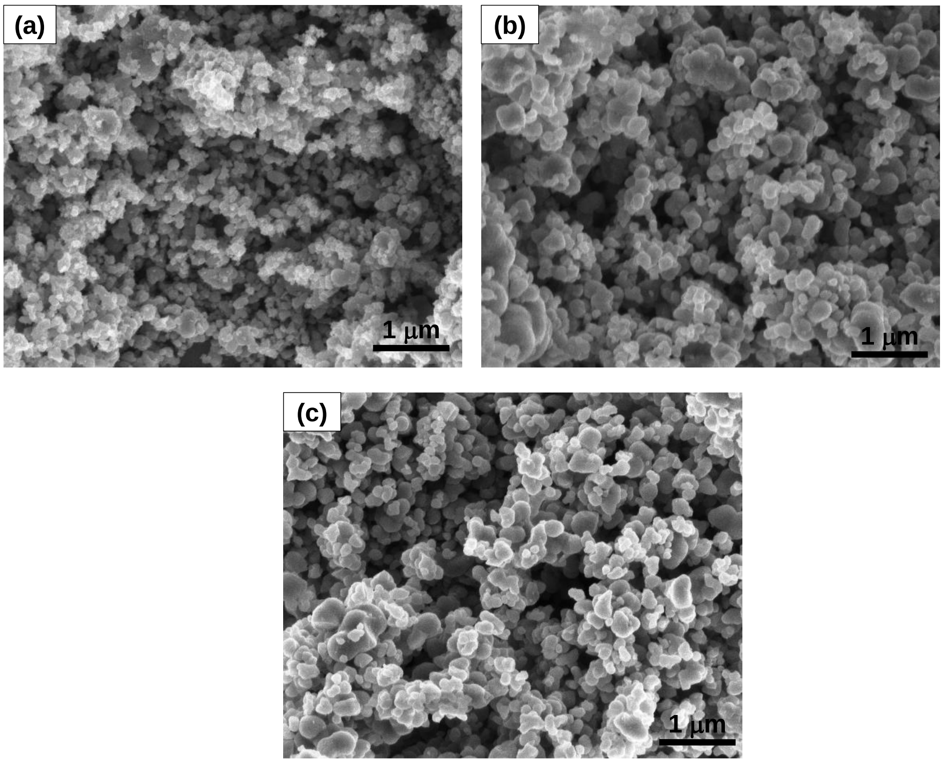


**Figure S3.** FESEM images of (a) 100T, (b) 100Z and (c) 25T75Z

Table S1

**Table S1.** Phase present in 100T, 75T25Z, 50T50Z, 25T75Z and 100Z nanoparticles as obtained in XRD analysis

| Sample | Phase present |
|---|---|
| 100T | $TiO_2$ (anatase) |
| 75T25Z | Anatase, hexagonal zinc titanate ($ZnTiO_3$), $TiO_2$ (rutile) |
| 50T50Z | Cubic zinc titanate (c-$Zn_2Ti_3O_8$) |
| 25T75Z | c-$Zn_2Ti_3O_8$, ZnO (zincite) |

| 100Z | Zincite |
|---|---|

Table S2

**Table S2.** At% of Zn an Ti of the corresponding metal oxide

| **Metal oxide** | **At%** | |
|---|---|---|
| | **Zn** | **Ti** |
| **100T** | 0 | 33.01 |
| **75T25Z** | 11.13 | 32.23 |
| **50T50Z** | 20.09 | 23.11 |
| **25T75Z** | 31.11 | 10.48 |
| **100Z** | 52.03 | 0 |
